# Intervertebral Disc Tissue Engineering with Natural Extracellular Matrix-Derived Biphasic Composite Scaffolds

**DOI:** 10.1371/journal.pone.0124774

**Published:** 2015-04-20

**Authors:** Baoshan Xu, Haiwei Xu, Yaohong Wu, Xiulan Li, Yang Zhang, Xinlong Ma, Qiang Yang

**Affiliations:** 1 Department of minimally invasive spine surgery, Tianjin Hospital, 406 Jie Fang Nan Road, Hexi District, Tianjin, 300211, People’s Republic of China; 2 Tianjin medical university, Tianjin, 300070, People’s Republic of China; 3 Cell Engineering Laboratory of Orthopaedic Institute, Tianjin Hospital, Tianjin, 300211, People’s Republic of China; University of Rochester, UNITED STATES

## Abstract

Tissue engineering has provided an alternative therapeutic possibility for degenerative disc diseases. However, we lack an ideal scaffold for IVD tissue engineering. The goal of this study is to fabricate a novel biomimetic biphasic scaffold for IVD tissue engineering and evaluate the feasibility of developing tissue-engineered IVD *in vitro* and *in vivo*. In present study we developed a novel integrated biphasic IVD scaffold using a simple freeze-drying and cross-linking technique of pig bone matrix gelatin (BMG) for the outer annulus fibrosus (AF) phase and pig acellular cartilage ECM (ACECM) for the inner nucleus pulposus (NP) phase. Histology and SEM results indicated no residual cells remaining in the scaffold that featured an interconnected porous microstructure (pore size of AF and NP phase 401.4±13.1 μm and 231.6±57.2 μm, respectively). PKH26-labeled AF and NP cells were seeded into the scaffold and cultured *in vitro*. SEM confirmed that seeded cells could anchor onto the scaffold. Live/dead staining showed that live cells (green fluorescence) were distributed in the scaffold, with no dead cells (red fluorescence) being found. The cell—scaffold constructs were implanted subcutaneously into nude mice and cultured for 6 weeks *in vivo*. IVD-like tissue formed in nude mice as confirmed by histology. Cells in hybrid constructs originated from PKH26-labeled cells, as confirmed by *in vivo* fluorescence imaging system. In conclusion, the study demonstrates the feasibility of developing a tissue-engineered IVD *in vivo* with a BMG- and ACECM-derived integrated AF-NP biphasic scaffold. As well, PKH26 fluorescent labeling with *in vivo* fluorescent imaging can be used to track cells and analyse cell—scaffold constructs *in vivo*.

## Introduction

Intervertebral-disc (IVD) degeneration usually causes chronic low back pain and other clinical symptoms, which will greatly reduce the quality of life of patients [1–3]. Current treatments for diseases resulting from IVD degeneration, including both conservative treatments and invasive procedures, cannot restore disc structure and function [4]. Therefore, developing effective therapies for IVD degeneration is urgently needed. Tissue-engineering techniques, by replacing the damaged IVD with scaffolds and appropriate cells, have emerged as a promising therapeutic approach to treat DDD [5].

The scaffold, as a major component in tissue engineering, provides space for cell proliferation and accumulation of extracellular matrix (ECM). It is supposed to support physiological load *in vivo* instead of damaged tissue. So, an ideal scaffold is essential in IVD tissue engineering; it should have good biocompatibility, moderate porosity and be similar to native IVD in shape, structure and mechanical properties [6].

IVDs are composed of 2 distinct anatomic regions: an outer ring (annulus fibrosus, AF) and an inner core (nucleus pulposus, NP), with a transitional zone that merges these 2 regions together. AF is a multi-lamellar fibro-cartilagenous ring composed of types I and II collagen, large aggregating proteoglycans, and fibroblast-like cells. Type I collagen accounts for nearly 70% of the dry weight of the outer AF, with type II collagen gradually increasing and type I collagen decreasing from the outer to inner AF [7]. Each layer of the AF has an oriented collagen architecture, with adjacent lamellae alternating in fiber angles approximately 30° to the transverse plane of the disc [8]. With this unique structure, AF provides powerful tensile strength to keep the NP in its position. The NP is a gelatinous structure, composed primarily of type II collagen, large aggregating proteoglycans, and a low concentration of chondrocytes. The NP can retain large amounts of water to provide resistance to compression.

Researchers have attempted to construct AF scaffolds or NP scaffolds in isolation with different materials, such as poly-L-lactic acid (PLLA), collagen, atelocollagen, silk, alginate, chitosan, collagen-glycosaminoglycan, and collagen/hyaluronan [9–16]. However, IVD degeneration usually involves both outer AF and central NP, which need to be repaired simultaneously to restore the function of IVD. So composite AF and NP scaffold is indispensable, and some researchers have had some success in this area. Park et al. [17] constructed a composite IVD scaffold with silk protein for the AF and fibrin/hyaluronic acid (HA) gels for the NP. The outer phase of the scaffold was seeded with porcine AF cells to form AF tissue, whereas chondrocytes were encapsulated in fibrin/HA hydrogels for the NP tissue and embedded in the center of the toroidal disk. After culture for 6 weeks, IVD containing both AF and NP tissue was formed *in vitro*. Lazebnik et al. [18] developed a biomimetic IVD by electrospinning circumferentially orientated polycaprolactone fibres (AF analogue) seeded with porcine chondrocytes and gelling a cell—agarose solution in the centre (NP analogue). However, these composite scaffolds had a severe defect: definite boundaries between outer and central materials of the scaffold, which did not mimic the transition zone of native IVD.

Moreover, the materials used for IVD scaffolds are usually a single material, synthetic or derived from ECM, which greatly differs from the ECM of natural IVD, composed of type I and II collagen, proteoglycans and various growth factors. Acellular ECM is a new source of scaffold material that has attracted much attention. It has good biocompatibility, contains various ECM components and growth factors that can promote cell proliferation and differentiation. Acellular ECM may be an ideal candidate for a scaffold in the construction of tissue-engineered IVD. In recent years, some kinds of acellular ECM used as scaffolds for IVD tissue engineering have included small intestinal submucosa (SIS) [19], acellular nucleus pulposus [20], and demineralized bone matrix gelatin (BMG) [4], with satisfactory results. Demineralized BMG contains large amounts of collagen I and proteoglycans, similar to the AF in composition. Acellular cartilage ECM (ACECM) contains large amounts of collagen II and proteoglycans, similar to the natural NP in composition.

Here, we attempted to construct a novel IVD scaffold with demineralized BMG (outer AF phase) and ACECM (inner NP phase) for IVD tissue engineering. We report on constructing tissue-engineered IVD with the novel scaffold and IVD cells, transplanting the cell—scaffold constructs subcutaneously into nude mice and evaluating the cells by fluorescent dye PKH26 and *in vivo* fluorescence imaging.

## Materials and Methods

### 1. Fabrication of the biphasic scaffold

#### 1.1 Preparing the AF phase of biphasic scaffold

**Fig 1:**
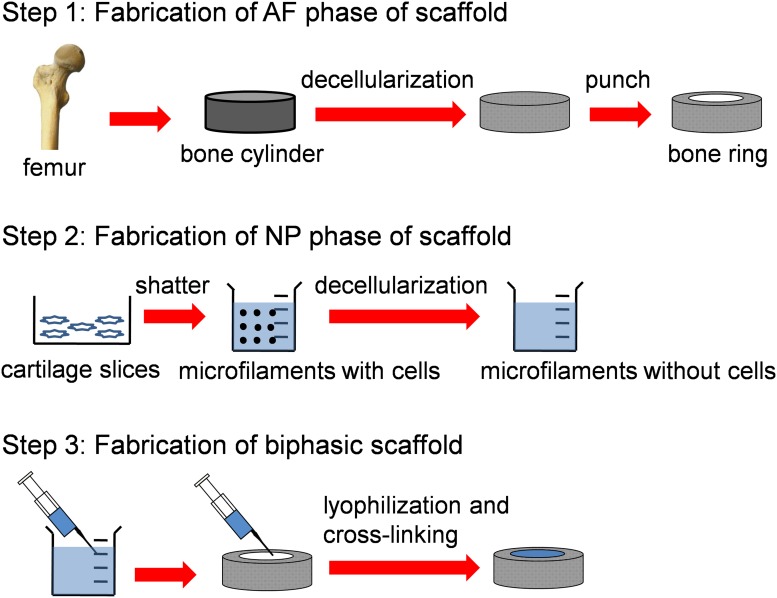
The biphasic scaffold fabrication process.

All animals used in this study were obtained from Animal Experimental Room of Tianjin Hospital. All animal experiments were approved by the Animal Experimental Ethics Committee of Tianjin Hospital and the animals were treated according to the experimental protocols under its regulations. The biphasic scaffold was fabricated as schematic diagram (Fig 1). Briefly, femurs were harvested aseptically from 6 adult pigs (large white pig, 6 months old, 3 males) within 6 h after they were killed. Muscle and ligaments were removed from the femurs before cancellous bone cylinders (10 mm diameter, 3-mm thick) were obtained from proximal or distal porcine femurs by use of a circular saw. After the marrow tissues were removed with sterile deionized water, the specimens were demineralized at 4°C with 0.6 M hydrochloric acid overnight; decellularized with 5% TritonX-100 for 12 h; washed with 2 M CaCl_2_ for 1 h at 4°C and 0.5 M ethylenediamine tetraacetic acid (EDTA, Sigma, USA) for 1 h at 4°C [21]; and washed with 8 M LiCl for 1 h. Subsequently the cylinder was shaped into a hollow ring with a 5-mm internal diameter by use of a punch.

#### 1.2 Preparing the NP phase of the biphasic scaffold

The inner NP phase was made of ACECM. Cartilage slices cut from caput femoris and femoral condyle of 10 pigs (large white pig, 6 months old, 5 males) were washed and shattered in phosphate buffered saline (PBS) containing 3.5% (w/v) phenylmethyl sulfonylfluoride (Merck, Germany) and 0.1% (w/v) EDTA. Cartilage microfilaments with diameters of approximately 500 nm to 5 μm were prepared by differential centrifugation, decellularized in 1% TritonX-100 for 12 h at 4°C, then in 50 U/mL deoxyribonuclease I and 1 U/mL ribonuclease A (both Sigma, USA) for 12 h at 37°C. Finally, microfilaments were washed with PBS and adjusted to a 3% (w/v) suspension [22].

#### 1.3 Preparing the biphasic scaffold

The 3% ACECM suspension was injected into the center of the AF phase and frozen at -80°C for 1 h. Finally, the biphasic scaffold was lyophilized and cross-linked with 14 mM ethyl-dimethyl-amino-propyl carbodiimide (EDAC, Sigma, USA) and 5.5 mM N-hydroxysuccinimide (NHS, Sigma, USA) for 24 h at 37°C. Excess EDAC and NHS were rinsed out of the scaffolds with PBS. The scaffolds were then sterilized by ^60^Coγ-irradiation (at 5 mrad).

### 2. Characterization of the biphasic scaffold

#### 2.1 Histology

Scaffolds were fixed in 10% (v/v) neutral buffered formalin, dehydrated and embedded in paraffin wax. They were cut into sections 10 μm thick by use of a microtome, and stained with haematoxylin and eosin (H&E) and Sirius red.

#### 2.2 SEM

Microstructures of cross sections of scaffolds were assessed by SEM (X-650, Hitachi, Japan). The pore sizes of the AF and NP phases were measured in 6 specimens (randomly selected 5 fields per specimen and 3 pores per field).

#### 2.3 Porosity and rehydration analysis

Porosity was calculated by liquid displacement methods [23]. Briefly, AF and NP phases in scaffold sections were separated with use of a blade, separately immersed in ethanol of V1 volume. AF or NP phase immersed in ethanol was pressed until no air was in the scaffold, leaving the pores of AF or NP phase filled with ethanol, then the volume of ethanol was recorded as V2. The scaffold pieces were removed from the ethanol and the volume of remaining ethanol was recorded as V3. Porosity was calculated as: (V1–V3)/(V2–V3)×100%.

The swelling ratio was calculated as follows: the scaffold was immersed in PBS for 24 h to achieve a fully hydrated state and weighed as Ww, then freeze-dried and weighed as Wd. The swelling ratio (%) was calculated as ((Ww-Wd)/Wd)×100%.

#### 2.4 Biomechanical properties

The dynamic mechanical performance test system EnduraTECELF 3200 (Bose, USA) was used for detecting biomechanical properties. Before testing, scaffolds were immersed in PBS (PH 7.4) for 2 h, then placed onto the loading platform under zero stress and compressed at a speed of 1 mm/min. The scaffold was kept moist during testing. All testing was conducted at room temperature and the elastic modulus was calculated. As the control, the elastic modulus of the native pig IVD tissue was performed.

#### 2.5 Toxicity of scaffolds

The toxicity of the biphasic scaffold that might result from the residual reagents and/or processing methods was detected by 3(4,5-dimethylthiazole-2-yl)-2,5-diphenyltetrazolium-bromide (MTT, Sigma, USA) assay. Scaffold extracts were obtained as follows [5]: scaffolds were minced and incubated in Dulbecco’s Modified Eagle’s Medium (DMEM) (3 ml/mg) at 37°C for 72 h with agitation. After incubation, the mixture was centrifuged (500 g, 15 min), and the supernatant was collected and supplemented with 10% (v/v) FBS, 100 U/ml streptomycin, 100 mg/ml penicillin. As a negative control, standard control medium was prepared in a similar way, except for omitting the diced scaffolds. Dimethyl sulfoxide (DMSO, Sigma, USA) was used as a positive control. Pig NP cells were prepared at 5×10^3^ cells/mL and seeded onto wells of 96-well plates (200 μl for each well). After 24 h incubation, the medium was replaced with standard control medium, DMSO and different concentrations (25%, 50%, 100%) of scaffold extracts. The plates were then incubated up to 7 days at 37°C in 5% (v/v) CO_2_, and the proliferation activity of the cells was determined by MTT assay every day. The optical density (OD) absorbance at 570 nm was determined with use of a microplate reader (RT-6000, Rayto, USA).

### 3. *In vitro* studies

#### 3.1 Isolation and culture of cells

Lumbar spines were dissected aseptically from pig, and AF and NP tissue was separated from IVDs with use of a blade, cut into small pieces separately. AF tissue was digested with 0.2% collagenase II (Sigma, USA) for 8 h and NP tissue was digested with 0.2% collagenase II for 4 h, respectively, at 37°C with 5% CO_2_. Cell suspensions were cultured in DMEM containing 10% fetal bovine serum (FBS, Gibco, USA) and 1% antibiotics at 37°C in a humidified atmosphere of 5% CO_2_. Cells at passage 1 were used for the following studies.

#### 3.2 Cell labeling

AF and NP cells were labeled with the fluorescent dye PKH26 (Sigma, USA). Briefly, cells were digested with trypsin to obtain single-cell suspensions, then washed with serum-free medium twice and resuspended in 1 mL of the dilution buffer C from the manufacturer’s labeling kit. The cell suspension was mixed with an equal volume of the labeling solution containing 4×10^-6^ M PKH26 in the dilution buffer and incubated for 5 min at room temperature. The reaction was terminated by adding 2 mL FBS. Cells were washed 3 times and prepared at 2×10^7^/mL for use. To determine whether PKH26 labeling affects proliferation of cells *in vitro*, the proliferation of PKH26-labeled and-unlabeled NP cells were simultaneously detected by MTT testing.

#### 3.3 Preparation of implants

Before inoculation, sterile scaffolds were rinsed with DMEM for 48 h. The liquid on the surface of scaffolds was dried by use of sterile filter paper. Then AF and NP cells were separately seeded into the corresponding phase of the biphasic scaffold at 2×10^7^ cell/mL (50 μl AF cells; 25 μl NP cells) by drop-wise addition onto the surface of the scaffold. At 1 h later, the scaffold was turned over and another cell suspension (50 μl AF cells; 25 μl NP cells) was seeded into the corresponding phase of the biphasic scaffold. The constructs were cultured for 2 h before being immersed in complete medium to allow cell attachment. Medium of 10 μl was added to the cell—scaffold construct every 30 min in case of cell death. The constructs were incubated *in vitro* at 37°C in 5% CO_2_, then implanted in nude mice after 2 days.

#### 3.4 Cell attachment and viability assessment

Cell attachment was evaluated by H&E staining and SEM after 48 h of *in vitro* culturing. H&E staining was performed as above. SEM was conducted as follows: briefly, samples were washed with PBS and fixed with 2.5% glutaraldehyde for 24 h at 4°C, dehydrated in a graded ethanol series to 100% ethanol, treated with hexamethyldisilazane, and sputter-coated with goldepalladium before viewing.

The viability of cells attached to the scaffold was evaluated by a live/dead assay kit (Molecular Probes, USA). Briefly, constructs were cut into sections of 100 μm by a microtome (RM2245, Leica, Germany) and incubated in 2 μM calcein AM (staining live cells) and 4 μM EthD-1 (dead cells) for 30–50 min, then washed and observed by confocal microscopy (TCS SP5 II, Leica, Germany).

#### 3.5 Real-time quantitative PCR

Implants were separated into AF and NP phases and treated with Trizol reagent (Invitrogen, USA) separately to isolate the cellular RNA. In total, 5 μg RNA was transcribed into cDNA by reverse transcriptase. Real-time quantitative PCR involved the SYBR-Green PCR Master mix (Applied Biosystems, USA), with a denaturation step for 4 min at 94°C, followed by 40 cycles for 30 s at 94°C, 60 s at 56°C and 40 s at 72°C. Gene expression was normalized to that of β-actin. Sequences of primers were for β-actin (5′AGGTTGGCTCTGACTGT3′, 5′TCTCCTTAATGTCACGCACG3′); collagen I (5′AGGGAGTTTACAGGAAGCAGACA3′, 5′CGAATACAAAACCACCAAGACC3′); collagen II (5′TTCAGCTATGGAGATGACAATC3′, 5′AGAGTCCTAGAGTGACTGAG3′); and aggrecan (5′GAGAT GGAGGGTGAGGTC3′, 5′ACGCTGCCTCGGGCTTC3′). As the control, real-time quantitative PCR of the native pig AF and NP tissue was performed.

### 4. *In vivo* studies

#### 4.1 Implantation

Four female nude mice (6 weeks old) were anesthetized with 10% chloral hydrate (0.3 mL/100 g weight). The backs of mice were disinfected before cell—scaffold constructs were implanted in each of 2 dorsal subcutaneous pockets created in each mouse. After implantation, the skin was closed and disinfected.

#### 4.2 Fluorescent imaging

After scaffolds were cultured for 6 weeks *in vivo*, fluorescent images of PKH26-labeled tissue-engineering IVD were acquired by Xenogen IVIS-200 Optical In Vivo Imaging System (Caliper, China). Before imaging, nude mice were anesthetized and placed in the right lateral position. The imaging parameters were exposure time, 2 min; F-stop, 4; FOV, 100 mm; resolution, 72 dpi; and optical density, >4.8. At the end of the imaging, mice were killed, and specimens were harvested and frozen immediately on dry ice.

#### 4.3 Histology

Specimens were mounted by use of O.C.T. compound (Tissue-Tek, Miles, USA). They were cut into cryosections 10 μm thick and stained with Safranin O and collagen I and II immunohistochemistry for observation of cell proliferation and substrate secretion *in vivo*. The process of collagen I and II immunohistochemistry was as follows: the cryosections were washed with phosphate-buffered saline (PBS), inactivated for endogenous peroxidase with 3% hydrogen peroxide in PBS for 15 min at room temperature, and blocked with 1% bovine serum albumin in PBS for 30 min to prevent nonspecific binding of proteins. Subsequently, the cryosections were incubated with monoclonal anti-collagen I or II antibody (Abcam, UK) diluted 1:100 in PBS at 4°C overnight in a humidified chamber, and then stained with goat anti-mouse FITC-conjugated secondary antibody (Abcam, UK) for 2 h at room temperature. Slides were then treated with 3,3'-diaminobenzidine (DAB, Sigma, USA) for approximately 10–15 min until color developed. Stained cryosections were observed by inverted microscope (TS100, Nikon, Japan).

### 5. Statistics

All data were analyzed using SPSS 16.0 software (SPSS, Chicago, IL, USA). Results were expressed as Mean ± SD. Differences between groups were assessed using one-way analysis of variance, followed by Sceffe or Tamhane’s T2 tests for multiple comparisons. A value of P < 0.05 was considered statistically significant.

## Results

### 1. Characterization of the biphasic scaffold

**Fig 2:**
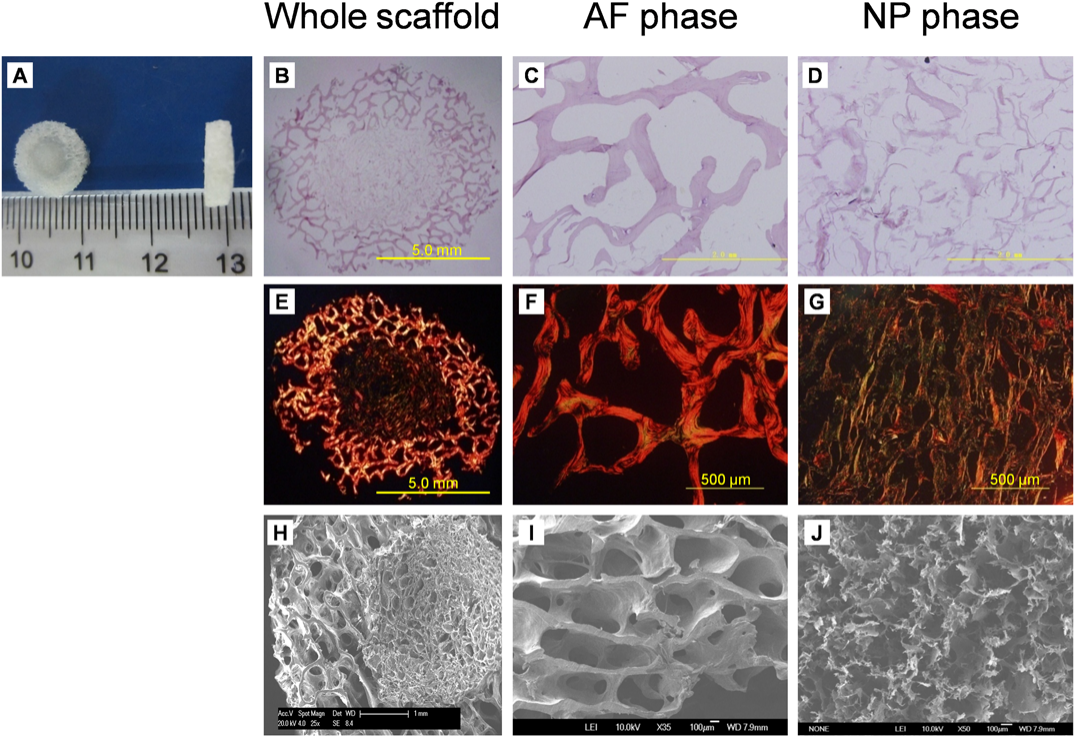
The macroscopic and microscopic structure of biphasic scaffolds. (A) Representative macroscopic images of biphasic scaffolds. The biphasic scaffold was white and looked similar to native IVD in shape. (B-D) H&E staining of cross-sections of biphasic scaffolds. Cells were removed from both AF and NP phases of the scaffold. (E-G) Sirius red staining of cross sections of biphasic scaffolds. AF phase stained intensive red, representing collagen I, and NP phase stained red or green, representing collagen II. (H-J) SEM of cross sections of biphasic scaffolds. Good integration between the AF and NP phases could be viewed. Both AF and NP phases possessed an interconnected porous structure.

**Fig 3:**
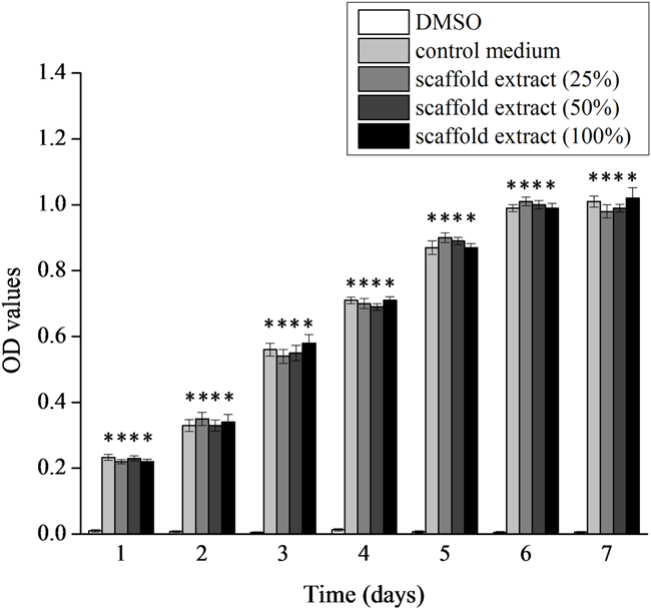
Cytotoxicity of biphasic scaffolds. MTT assay of proliferation of NP cells cultured with different concentrations of scaffold extracts. The proliferation rate of cells cultured with DMSO was significantly lower than those of the other groups (P<0.05). However, there was no significant difference in cell proliferation among cells cultured with control medium and different concentrations of scaffold extracts at the same culture time (P>0.05). * = p<0.05 compared to cells cultured with DMSO.

The disc-shaped biphasic scaffold looks white macroscopically, there being no crack between AF phase and NP phase (Fig 2A). H&E staining revealed that the cells were completely removed from both AF and NP phases of the scaffold (Fig 2B–2D), the immunogenicity of the scaffold being eliminated. Sirius red staining showed the AF phase stained intensive red and the NP phase stained a variety of colors, including red, bright white, green, and dark grey, with the reticular collagen fibers loosely distributed (Fig 2E–2G), which indicated that the AF phase contained more collagen I and the NP phase contained more collagen II. SEM revealed good integration between the AF and NP phases (Fig 2H–2J), which is ideal for the formation of tissue-engineered integrated IVD. The AF phase possessed regular porous structure with porosity 63.6% and mean pore size 401.4±13.1 μm. The NP phase also showed an interconnected porous structure. The mean pore size was 231.6±57.2 μm and porosity 89.3%. The biphasic scaffold had a swelling ratio of 655.7±78.6%. The compressive modulus of the biphasic scaffold was lower than that of native disc (49.1±15.6 kPa vs 135.9±28.9 kPa) (P<0.05). MTT assay revealed that the proliferation rate of cells cultured with DMSO was significantly lower than those of the other groups (P<0.05). However, there was no significant difference in cell proliferation among cells cultured with control medium and different concentrations of scaffold extracts at the same culture time (P>0.05) (Fig 3), from which we can infer that scaffold extracts had no influence on cell proliferation and the scaffold is non-toxic.

### 2. *In vitro* studies

#### 2.1 IVD cell culture and PKH26 labeling

**Fig 4:**
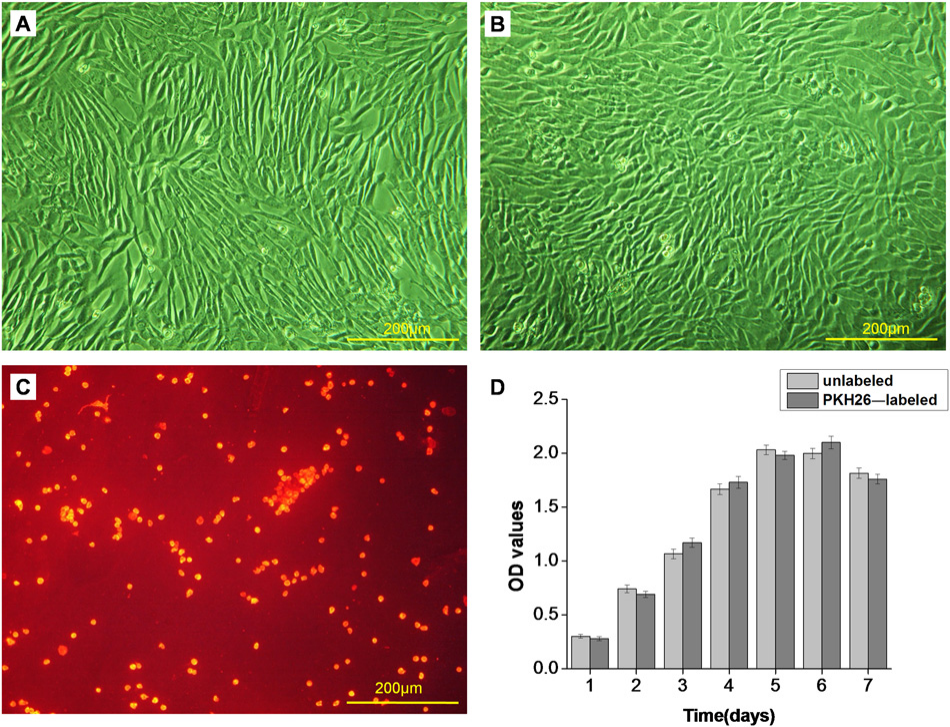
The morphology of AF cells, NP cells and PKH26-labeled NP cells and the proliferation of PKH26-labeled NP cells. (A) Optical microscopy of the morphology of AF cells. AF cells showed a long spindle-like morphology. (B) Optical microscopy of the morphology of NP cells. NP cells showed a short spindle-like or oval morphology. (C) Fluorescence microscopy of PKH26-labeled NP cells. The labeled cells displayed uniformly red fluorescence. (D) MTT assay of the proliferation of PKH26-labeled and-unlabeled NP cells. There was no significant difference in cell proliferation between PKH26-labeled and-unlabeled NP cells at the same culture time (P>0.05).

Optical microscopy revealed the adherent AF cells with a long spindle-like morphology and NP cells with a short spindle-like or oval morphology (Fig 4A and 4B). AF cells and NP cells were all labeled by PHK26, and the labeled cells displayed uniformly red fluorescence. The AF cells labeled with PKH26 were similar with NP cells labeled with PKH26 under fluorescence microscopy, so we showed the image of PKH26-labeled NP cells for represent (Fig 4C), from which it could be found that PKH26 had a labeling rate of 100%. MTT assay revealed no significant difference in cell proliferation between PKH26-labeled and-unlabeled NP cells at the same culture time (P>0.05), which indicated that PKH26 had no influence on cell proliferation (Fig 4D).

#### 2.2 Cell attachment and viability

**Fig 5:**
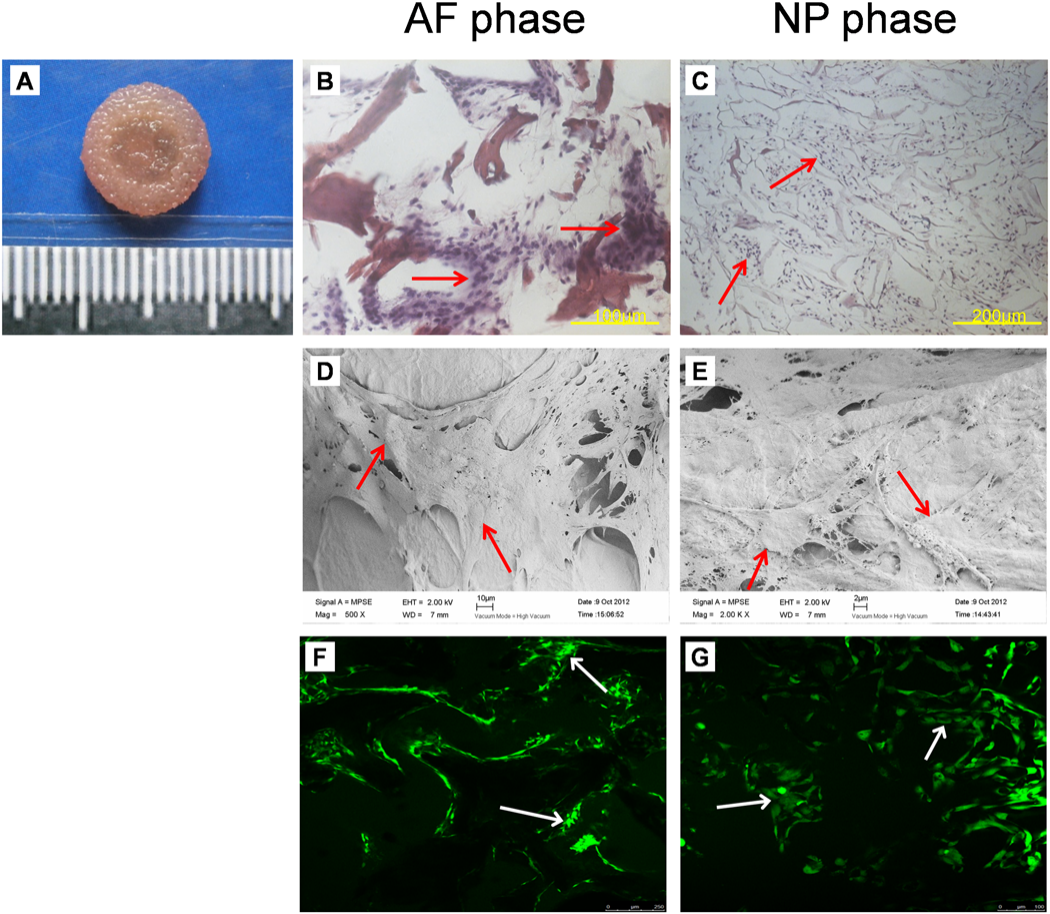
The macroscopic and microscopic observation of cell—scaffold constructs cultured for 48-h *in vitro*. (A) Macroscopic images of cell—scaffold constructs cultured for 48-h *in vitro*. The constructs appeared pink. (B-C) H&E staining of cross sections of constructs. Both AF and NP phases were filled with large amounts of cells (arrows), indicating that cells had a good proliferation in the scaffold. (D-E) SEM of cells in constructs. Both phases were filled with cells (arrows) and large amounts of ECM. (F-G) Live/dead staining of cells in constructs. Cells (arrows) in the constructs displayed green fluorescence, with no red flurescence. (green fluorescence: viable, red fluorescence: necrotic).

After 48-h incubation, cell—scaffold constructs appeared pink (Fig 5A). H&E staining showed both AF and NP phases filled with large amounts of cells, which covered most of the pores (Fig 5B and 5C). Cells in the scaffold were surrounded by large amounts of collagen fibers and ECM on SEM (Fig 5D and 5E). By live/dead staining, cells in the cell—scaffold constructs showed green fluorescence (live cells), with no red fluorescence (dead cells) (Fig 5F and 5G).

#### 2.3 Real-time quantitative PCR of gene expression in AF and NP cells

**Fig 6:**
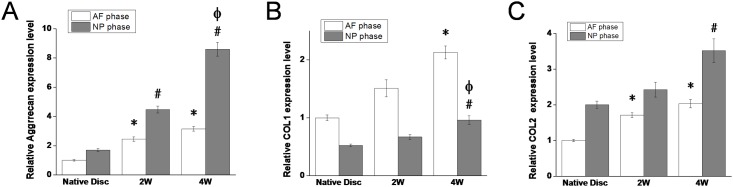
RT-PCR analysis of gene expression of aggrecan (A), collagen I (B) and II (C) in AF cells and NP cells in cell—scaffold constructs and native AF/NP. * = p<0.05 compared to native AF, # = p<0.05 compared to native NP, Φ = p<0.05 compared to NP phase of 2W.

The gene expression of aggrecan and collagen I and II of AF and NP cells in the implants were all increased with time. After culture for 4 weeks, the gene expression in AF cells was 3.15-, 2.13- and 2.03-fold that of native AF and in NP cells was 5.05-, 1.83- and 1.76-fold that of native NP (Fig 6).

### 3. *In vivo* studies

#### 3.1 Fluorescent imaging

**Fig 7:**
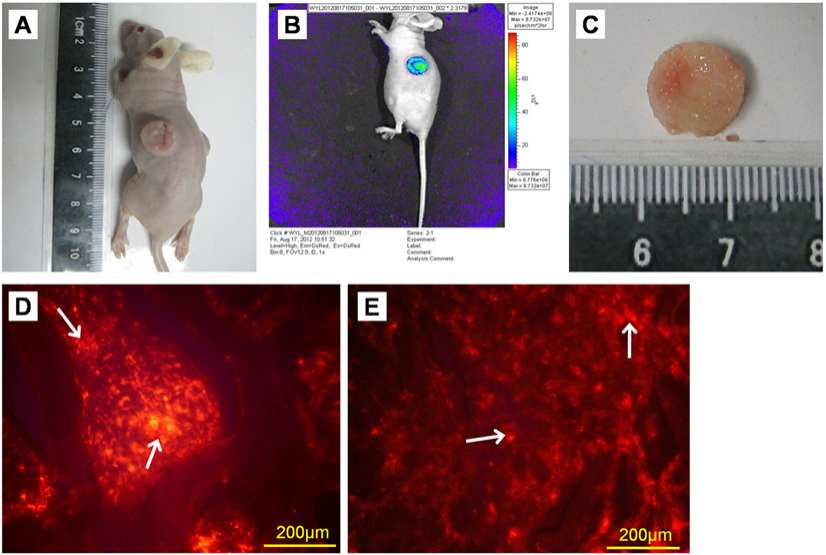
The fluorescent imaging of PKH26-labeled cell—scaffold constructs cultured for 6 weeks in dorsal subcutaneous pockets created in nude mice. (A) Macroscopic image of the locations of implanted labeled cell—scaffold constructs in nude mice. There was no infection at the implantation position. (B) *In vivo* fluorescence imaging of cell—scaffold constructs implanted subcutaneously in nude mice. Fluorescence was evenly distributed in the constructs, nowhere else of nude mice. There was a color bar on the right, which represented the cell density in the constructs. From top to bottom of the color bar, red indicated a relative high cell density and blue represented a relative low cell density. (C) Macroscopic image of cell—scaffold construct retrieved from nude mice after culture for 6 weeks *in vivo*. The construct was deep yellow and similar to natural IVD in shape and tenacity, with AF and NP phases closely connected. (D-E) Fluorescence microscopy of cell—scaffold constructs. Both AF phase (D) and NP (E) phase were full of cells with red fluorescence (arrows).

Cell—scaffold constructs implanted subcutaneously in nude mice were imaged over 6 weeks. There was no infection at the implantation position (Fig 7A). *In vivo* fluorescent images of constructs showed fluorescence evenly distributed in the constructs, nowhere else of nude mice, indicating PKH26-labeled cells proliferated within the scaffold and did not move out of the scaffold (Fig 7B). From the color bar on the right of the picture, it can be found that NP phase possessed a slightly higher cell density than AF phase, which may be due to the relative small pore size of the NP phase scaffold as compared with the AF phase.

#### 3.2 Histology

**Fig 8:**
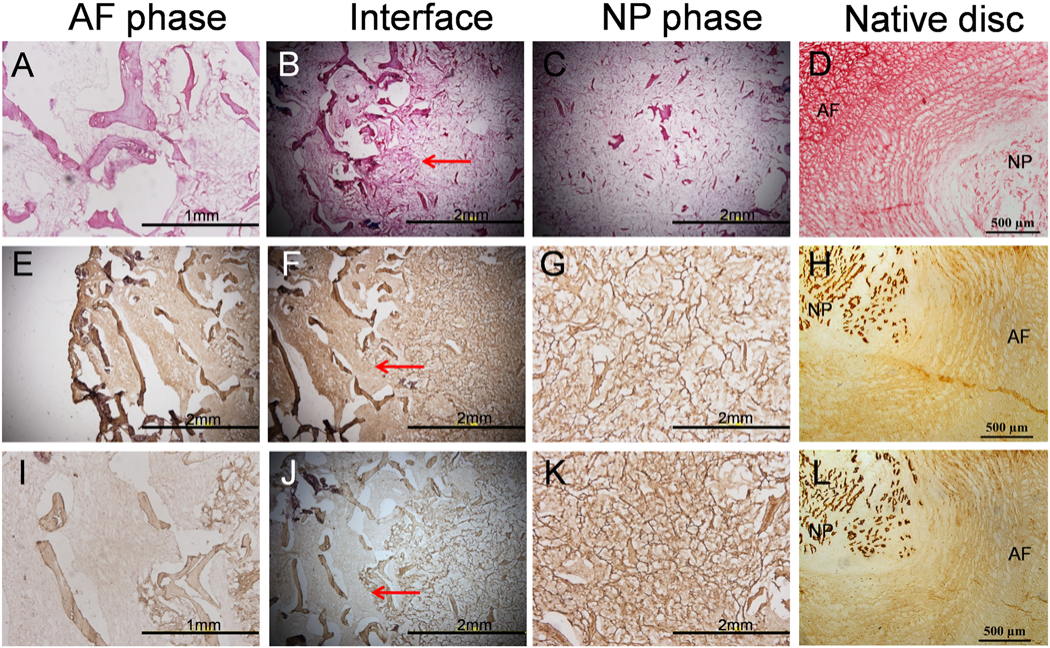
The histological staining of cell—scaffold constructs cultured for 6 weeks in dorsal subcutaneous pockets created in nude mice. (A-C) Safranin O staining of cross sections of cell—scaffold constructs. Safranin O was positive for both AF and NP phases, indicating the presence of ECM rich in sulfated proteoglycan. (D) Safranin O staining of cross sections of native pig disc, with both AF and NP positive. (E-G) Immunohistochemistry of collagen I in constructs. Immunohistochemistry of collagen I was intensively positive for both AF and NP phases. (H) Immunohistochemistry of collagen I in native pig disc. (I-K) Immunohistochemistry of collagen II in constructs. Immunohistochemistry of collagen II was positive for the NP phase and weakly positive for the AF phase. (L) Immunohistochemistry of collagen II in native pig disc. AF and NP phases of scaffold were closely connected after culture for 6 weeks *in vivo* (arrows indicating interface).

Specimens were retrieved from the subcutaneous pockets of sacrificed nude mice after culture for 6 weeks *in vivo*. The constructs were deep yellow and similar to natural IVD in shape and tenacity. AF and NP phases were closely connected, with invisible boundaries (Fig 7C). Red fluorescent cells labeled with PKH26 could be seen in most areas of the constructs (Fig 7D and 7E). All specimens could be stained with Safranin O, which indicates the presence of ECM rich in sulfated proteoglycan (Fig 8A–8C). Immunohistochemistry for collagen I was intensively positive for both AF and NP phases (Fig 8D–8F) and collagen II was positive for the NP phase and weakly positive for the AF phase (Fig 8G–8I).

## Discussion

In the present study, we fabricated a novel biomimetic biphasic scaffold for IVD tissue engineering with BMG and ACECM. The novel scaffolds had good biocompatibility and provided a suitable 3-D environment to support cell adhesion and proliferation. The cells seeded onto the scaffold could secrete large amounts of ECM *in vitro* and *in vivo* as confirmed by histology, so the scaffold could be used as an alternative scaffold for IVD tissue engineering.

In recent years, some researchers have tried to construct composite tissue-engineered IVDs. Mizuno et al. [24] constructed composite IVD structures consisting of AF and NP cells seeded on polyglycolic acid and calcium alginate matrices, respectively. The disc implants were placed in the subcutaneous space of the dorsum of athymic mice and harvested at 4, 8, and 12 weeks. Tissue-engineered AF was rich in type I collagen, but NP contained type II collagen, which increased in level with time, similar to the native disc at 12 weeks. Zhuang et al. [25] constructed tissue-engineered composite IVD consisting of demineralized BMG and collagen II/hyaluronate/chondroitin-6-sulfate (CII/HyA-CS) scaffolds seeded with AF and NP cells, respectively. The cell—scaffold hybrids were implanted in the subcutaneous space of the dorsum of athymic mice and harvested at 4, 8, and 12 weeks. Results revealed progressive tissue formation and junction integration between AF and NP regions. The content of DNA, proteoglycan and hydroxyproline increased with time and was similar to that in native tissue at 12 weeks. All these results demonstrated the feasibility of creating a tissue-engineered composite IVD with similar morphological and biochemical properties to the native tissue.

Many scaffolds, both natural and synthetic, have been used for IVD tissue engineering [26–31], but these scaffolds differ from natural IVD in components or structure. We fabricated and characterized a natural-origin, biphasic scaffold derived from 2 types of ECM: an outer BMG phase and inner ACECM phase. BMG contains large amounts of collagen I and proteoglycan, which are the main components of AF ECM. The BMG we used was derived from femurs, because they have a uniform pore size with a small coefficient of variation of the pore diameter (0.033). ACECM, which is rich in collagen II and proteoglycan and similar to NP ECM, is an ideal material for an NP scaffold. However, the native cartilage is so compact that the decellularization is ineffective, without adequate space for the growth and proliferation of seeded cells. So we prepared cartilage microfilaments by physical pulverization and differential centrifugation to achieve a completely decellularization and porous structure.

Constructing integrated IVD scaffold with a good combination of AF region and NP region has been a big challenge for researchers. Kim et al. constructed IVD scaffolds with demineralized bone particle (DBP) and poly (L-lactide-co-glycolide) (PLGA), however, the AF phase and NP phase were poorly connected, and there were obvious cracks between AF phase and NP phase [32]. We combined BMG and ACECM together by lyophilization and cross-linking agents (EDAC and NHS). The lyophilization technique can control the penetration depth of ACECM suspension into the outer BMG ring and combine ACECM with BMG. EDAC and NHS can help form collagen—collagen and collagen—GAG covalent links to strengthen the binding force. Yang et al. [22] succeeded in developing a 3-D interconnected porous cartilage scaffold derived from cartilage ECM through the freeze-drying and cross-linking techniques.

The novel scaffolds provide a suitable 3-D environment and appropriate pore size to support cell adhesion and proliferation. So far, scaffolds used for tissue engineered IVD differ in porosities and pore sizes. Scaffolds with porosities ranging from 75% to 97% and pore sizes ranging from 100 μm to 550 μm could provide a suitable environment for cells in constructing tissue engineering IVD [33–34]. In our study, the porosities of AF phase and NP phase are 63.6% and 89.3%, and the average pore sizes of AF phase and NP phase are (401.4±13.1) μm and (231.6±57.2) μm separately. The existing interconnected pores and proper pore size facilitate cell migration into internal pores, favor nutrients and metabolic waste transport.

Mechanical property is an important parameter of intervertebral disc. *In vivo* IVD serves to support large spinal loads, which are combinations of tension, torsion, compression, and bending. So, proper mechanical property of scaffold is essential for constructing tissue-engineered IVD which will be implanted into the body and function instead of the damaged IVD. The compressive modulus of the biphasic scaffold we fabricated was 49.1±15.6 kPa, lower than that of native disc (135.9±28.9 kPa). Still, the compressive modulus of the biphasic scaffold would gradually increase as cells are seeded into the scaffold and secret large amounts of ECM *in vivo* [35].

PKH26 belongs to lipophilic dyes that can irreversibly bind cell membranes and excite red fluorescence under a fluorescence microscope. When PKH26-labeled cells divide, the dye can be evenly distributed to the daughter cells. So PKH26 is widely used for cell tracing *in vivo*. As well, *in vivo* fluorescence imaging has been widely used to monitor tumor cell growth, metastasis and growth of transgenic cells. Recently some researchers began to use it in tissue engineering research and achieved satisfactory results. Yang et al. [22] labeled chondrogenically induced bone-marrow—derived mesenchymal stem cells with PKH26, then labeled cells were seeded into scaffolds and implanted subcutaneously into nude mice. Four weeks later, *in vivo* fluorescent imaging showed the site of constructs with intense fluorescence. In our study, the PKH26-labeled cell—scaffold constructs were implanted into subcutaneous pockets of nude mice, cultured for 6 weeks *in vivo* and detected by *in vivo* imaging system. *In vivo* fluorescence imaging of cell—scaffold constructs showed that fluorescence was evenly distributed in the constructs, nowhere else of nude mice, indicating that the seeded cells remained in the scaffold. We did not trace AF cells or NP cells alone to evaluate the distribution of AF or NP cells. It is also acceptable that some AF cells migrate into NP phase or some NP cells migrate into AF phase. The mutual migrate will form the transitional zone that merges AF and NP together.

As we know, native NP tissue does not have collagen I or possesses small amounts of collagen I. In our study, immunohistochemistry of collagen I was intensively positive for NP phase after culture for 6 weeks *in vivo*, which was thought to be related to the following three reasons. First, the NP cells seeded into the scaffold underwent the process of separation from native disc, culture and passage *in vitro*. During this process, the NP cells were treated with collagenase II, trypsin and other reagents, which maybe changed the biological characteristics of NP cells and increased the secretion of collagen I. Second, degeneration maybe happened in the NP cells during the culture *in vitro*, and the expression of collagen I increased. In our further study, stem cells will be applied instead of NP cells, because stem cells have a good stability and do not degenerate easily. Third, the AF phase and NP phase of the scaffold were closely connected and the pores of two phases were communicated with each other. Maybe some AF cells seeded into the AF phase of scaffold migrated into the NP phase during the culture and secreted large amounts of collagen I.

There are also limitations in our study. Mechanics plays an important role in the construction and remodeling of tissue-engineered IVD. The proliferation and gene expression of cells, accumulation of ECM and biomechanical properties will be influenced when the cell—scaffold constructs are cultured under forces. In this study, we did not present the study of cell—scaffold constructs cultured under different forms of force *in vitro* and *in vivo*. Further research will be directed to investigate the cell proliferation, ECM secretion and biomechanical properties of cell-scaffold constructs cultured under tension, torsion, compression and bending.

## Conclusion

The study demonstrates the feasibility of developing a tissue-engineered IVD *in vivo* with a BMG- and ACECM-derived integrated AF-NP biphasic scaffold. As well, PKH26 fluorescent labeling combined with *in vivo* fluorescent imaging are capable of tracking cells and analyzing cell—scaffold constructs *in vivo*.
